# **Long-term Outcomes of Children Undergoing Thoracotomy Lung Resection for** **Congenital Lung Malformations**

**DOI:** 10.1007/s00595-026-03242-y

**Published:** 2026-02-11

**Authors:** Marie Todo, Koichi Deguchi, Hiroomi Okuyama, Miho Watanabe

**Affiliations:** 1 Department of Pediatric Surgery, Graduate School of Medicine, Osaka University, 2-15 Yamadaoka, Suita, Osaka, 565－0871 Japan; 2 Department of Pediatric Surgery, Osaka City General Hospital, Osaka, Japan; 3 Department of Surgery, Tottori University Hospital, Tottori University, Tottori, Japan

**Keywords:** Congenital lung malformations, Long-term outcome, Lung function test, Musculoskeletal morbidity, HRQOL

## Abstract

**Purpose:**

We evaluated the long-term outcomes of children undergoing thoracotomy lung resection for congenital lung malformations in terms of lung function, complications, and health-related quality of life (HRQOL).

**Methods:**

We retrospectively reviewed 27 children who underwent thoracotomy at Osaka University Hospital (1992–2017) with at least five years of follow-up and postoperative lung function testing after six years of age. We compared the percent predicted vital capacity (%VC), percent predicted forced expiratory volume in 1 s (%FEV_1_), and FEV_1_ to FVC ratio (FEV_1_/FVC) as indicators of the lung function. Longitudinal changes in the lung function, pulmonary and musculoskeletal morbidities, and HRQOL were assessed using the Pediatric Quality of Life Inventory.

**Results:**

%VC and FEV_1_/FVC remained within the normal range but were significantly lower than the controls; %FEV_1_ was below the normal range. No significant longitudinal changes in the lung function were observed. Asthma‑like symptoms appeared in 37.0% of the patients and persisted beyond adolescence. Musculoskeletal morbidities occurred in 33.3% of the patients, with five pectus excavatum cases requiring correction. HRQOL did not differ from that of the healthy controls.

**Conclusions:**

In our study, children undergoing thoracotomy lung resection for congenital lung malformations exhibited a significantly lower lung function than the healthy controls, and these impairments persisted over an extended follow-up period. Long-term complications and a reduced lung function were not reflected in the subjective HRQOL.

**Supplementary Information:**

The online version contains supplementary material available at 10.1007/s00595-026-03242-y.

## Introduction

Congenital lung malformations comprise a heterogeneous group of developmental abnormalities including congenital pulmonary airway malformations (CPAM), bronchopulmonary sequestration (BPS), congenital lobar emphysema (CLE), bronchial atresia (BA), and bronchogenic cysts (BC). These disorders result from abnormal airway patterning and branching morphogenesis during fetal lung development, producing cystic or emphysematous malformed pulmonary tissue, sometimes accompanied by abnormal vascular structures [1, 2].

Although rare, congenital lung malformations are increasingly being detected prenatally due to advances in ultrasonography [3]. The clinical presentation ranges from asymptomatic lesions to severe manifestations, such as recurrent respiratory infections, neonatal respiratory distress, or fetal hydrops [4].

Although surgical resection generally alleviates respiratory symptoms and prevents complications, concerns remain regarding long-term functional and structural sequelae. The reported long-term respiratory outcomes vary; some studies indicate preserved lung capacity, whereas others describe reduced forced expiratory volume in 1 s (FEV_1_) and forced vital capacity (FVC) after surgery [5,6,7,8]. Musculoskeletal morbidities (particularly after thoracotomy) and asthma-like symptoms in children undergoing lung resection have been described; however, their prevalence, severity, and impact on daily functioning remain unclear. Moreover, previous studies comparing thoracotomy and thoracoscopic approaches have suggested potential advantages of thoracoscopic surgery but have reported inconsistent results regarding long-term pulmonary and musculoskeletal morbidities [6, 9], thus leaving the optimal surgical approach and its long-term implications a matter of ongoing debate.

Importantly, the associations between long-term morbidities (e.g., asthma-like symptoms and musculoskeletal morbidities) and lung function tests are lacking. Furthermore, little is known about how these sequelae influence exercise tolerance and health-related quality of life (HRQOL) through adolescence.

This study aimed to (i) evaluate the long-term impact of lung resection by thoracotomy on lung function in children with congenital lung malformations, (ii) identify factors associated with pulmonary impairment, and (iii) determine whether postoperative sequelae affect exercise tolerance or HRQOL.

## Methods

### Study design and ethical approval

This retrospective cohort study was conducted at the Osaka University Hospital and approved by the institutional review board (Approval No. 19507(T6)−2).

## Patient selection

Patients were eligible for inclusion if they:

1. had undergone surgical resection for congenital lung malformations (CPAM, BPS, BA, and unclassified cases) between January 1992 and December 2017.
2. had a histopathological confirmation of diagnosis; cases in which a definitive diagnosis could not be established were classified as “unclassified.”
3. had ≧ 5 years postoperative follow-up, and.
4. had at least one lung function test performed after the age of 6 years.

**Fig. 1:**
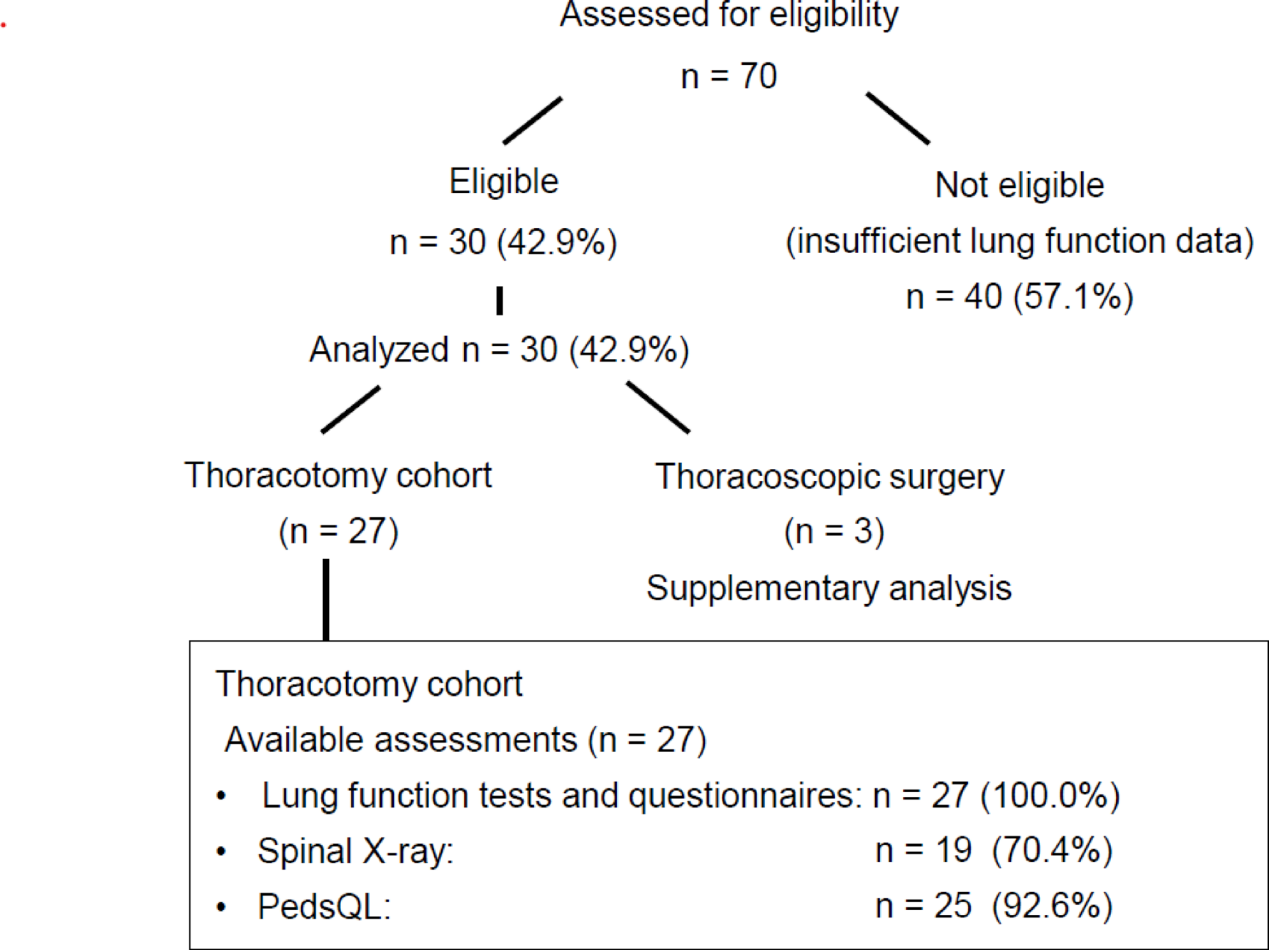
Flow Diagram of Participants**.** Patients who had undergone lung resection for congenital lung malformations were assessed for eligibility. Inclusion criteria were a follow-up period of at least 5 years and availability of postoperative lung function data after 6 years of age. Of the 30 eligible patients, 27 who underwent thoracotomy were included in the primary analysis, while 3 who underwent thoracoscopic surgery were excluded from the primary analyses and evaluated separately as supplementary cases. The thoracotomy cohort was analyzed for the lung function, pulmonary and musculoskeletal morbidities, and health-related QoL

Of the 70 eligible patients, 40 were excluded owing to a lack of lung function data, resulting in a total cohort of 30 patients (Fig. 1).

Primary analyses were performed in patients who underwent thoracotomy (*n* = 27), as only three patients underwent thoracoscopic surgery. Thoracoscopic cases are presented separately in the supplementary data.

## Data collection

Medical records were reviewed for demographic and clinical data, including sex, affected side, surgical approach, extent of resection, age at surgery, prenatal diagnosis, fetal interventions, and histopathologic subtype. Surgical extent was categorized as lobectomy, segmentectomy, partial resection (wedge or subsegmental), or combined lobectomy and partial resection.

In patients who underwent thoracotomy, additional surgical details, including the incision length and intercostal level, were recorded when available and explored descriptively as potential confounders of postoperative lung function and musculoskeletal outcomes.

Fetal intervention was not included in the multivariable analyses, as only one patient in the thoracotomy cohort had undergone this procedure.

## Lung function tests

Spirometry was performed according to American Thoracic Society/European Respiratory Society (ATS/ERS) guidelines. The following parameters were recorded.

- Percentage of predicted vital capacity (%VC).
- Percent predicted forced expiratory volume in 1 s (%FEV_1_).
- FEV_1_/FVC ratio.

The predicted values were calculated using the Global Lung Function Initiative (GLI) 2012 multi-ethnic reference equations [10].

Spirometry results from age-matched children with conditions unrelated to respiratory function were used as the controls. A reduced lung function was defined as %VC < 80%, %FEV_1_ < 80%, FEV_1_/FVC < 80%, or consistent with the conventional criteria for ventilatory impairment [11, 12].

To minimize the influence of the resection extent, a subgroup analysis was performed among patients who underwent anatomical single-lobe resection (*n* = 20).

In addition, longitudinal changes in %VC, %FEV_1_, and FEV_1_/FVC were analyzed.

## Symptoms and functional assessment

Asthma-like symptoms were evaluated using a questionnaire based on Expert Panel Report 3 of the National Asthma Education and Prevention Program [13], including residual cough, wheezing, allergic cough, and cough without an apparent trigger. Data on ongoing treatment, exercise status, and exertional dyspnea were also collected. Asthma-like symptoms and related clinical variables were analyzed as potential factors associated with the postoperative lung function.

### Musculoskeletal assessment

Postoperative musculoskeletal morbidities were evaluated through physical examination, chest radiography, and when indicated, computed tomography (CT) or spinal radiography. The morbidities were classified as follows:

- Pectus excavatum: Haller Index > 3.25 on CT,
- Scoliosis: Cobb angle ≥ 10° on spinal radiography (assessed in participants who consented, *n* = 19).
- Other chest wall deformities: unilateral or asymmetric lateral depression, unilateral shoulder elevation, or breast asymmetry.>

Musculoskeletal morbidities were analyzed both as long-term postoperative outcomes and potential factors associated with lung function. In addition, exploratory analyses were performed to assess the associations between musculoskeletal morbidities and thoracotomy-related factors, including skin incision length and intercostal level, when available.

## Quality of life assessment

Health-related quality of life (HRQOL) was assessed in 25 patients using the age-appropriate self-report and parent-proxy versions of the Pediatric Quality of Life Inventory (PedsQL™ Generic Core Scales), age-appropriate versions for self-report, and parent-proxy forms. Age-appropriate versions of the questionnaires were administered to minimize the potential impact of age differences between the groups on the survey results. Two patients were excluded because informed consent was not obtained. A total of 31 healthy children were recruited as controls after obtaining their informed consent.

### Statistical analysis

Analyses were performed using EZR version 4.3.1 (Saitama Medical Center, Jichi Medical University, Japan). Continuous variables are expressed as the mean ± standard deviation (SD) for normally distributed data and as median [interquartile range, IQR] for non-normally distributed data. Normality was assessed using the Shapiro–Wilk test. Categorical variables were expressed as numbers (percentages). Between-group comparisons were performed using Student’s *t-*test for normally distributed continuous variables with equal variances, Welch’s *t-*test for normally distributed variables with unequal variances, and Mann-Whitney U test for non-normally distributed continuous variables.

Univariate analyses examined the associations between lung function and perioperative variables, asthma-like symptoms, and musculoskeletal morbidities. In the univariate analysis, variables with *P* < 0.20 in univariate analysis, as well as clinically relevant covariates, were entered into a stepwise multiple linear regression model (backward elimination) to identify independent predictors of %VC, %FEV_1_, and FEV_1_/FVC. Model assumptions were assessed using residual and Q-Q plots, and multicollinearity was evaluated using variance inflation factors (VIFs). Statistical significance was defined as a two-sided P-value of < 0.05.

In patients who underwent thoracotomy, the association between postoperative lung function and skin incision length was evaluated using Spearman rank correlation coefficients. Differences in lung function across the intercostal levels of thoracotomy were assessed using the Kruskal–Wallis test.

The associations between musculoskeletal morbidities and skin incision length were examined using logistic regression analysis. Differences in the prevalence of musculoskeletal morbidities across intercostal levels were evaluated using Fisher’s exact test. Analyses of the skin incision length and intercostal level were considered exploratory because of the limited sample size.

## Results

### Patient characteristics

The primary analysis included 27 patients who underwent thoracotomy. Thoracoscopic cases (*n* = 3) were analyzed separately and are presented in Supplementary Table 1.

In the thoracotomy cohort, the study cohort included 16 males (59.3%) and 11 females (40.7%), with a median age of 12.0 [10.6–15.0] years at the last follow-up and a median follow-up period of 11.3 [9.7–14.1] years. The median age at surgery was 189.0 [75.0–350.5.0.5] days. Six patients (22.2%) underwent neonatal surgery, and prenatal diagnosis was made in 22 patients (81.5%) (Table 1).

**Table 1:** Patient Background and Surgical Treatments (n=27)

Sex, male		16	(59.3)
Age at surgery, days		189.0	(75.0–350.5)
Age at follow-up visit, years		12.0	(10.6–15.0)
Follow-up period, years		11.3	(9.7–14.1)
Age at surgery			
≤ 28days		6	(22.2)
> 28days		21	(77.8)
Prenatal diagnosis		22	(81.5)
Fetal interventions		1	(3.7)
Extent of resection			
Lobectomy or more	Lobectomy	20	(74.1)
	Lobectomy + Partial resection	1	(3.7)
Less than lobectomy	Segmentectomy	4	(14.8)
	Partial resection	2	(7.4)
Affected side, right		14	(51.9)
Data are presented as n (%) or the median (interquartile range, IQR).			

The surgical extent included lobectomy (*n* = 20, 74.1%), segmentectomy (*n* = 4, 14.8%), partial resection (*n* = 2, 7.4%), and combined lobectomy plus partial resection (*n* = 1, 3.7%). Fourteen lesions (51.9%) were right-sided.

One patient underwent a fetal intervention. The patient underwent fetal thoracentesis for a macrocystic lesion　at another institution. The patient was delivered at 33 weeks of gestation and underwent lobectomy on 1 day of life, followed by reoperation due to a persistent post operative air leak.

The histopathological diagnoses were CPAM Type 1 (*n* = 2), CPAM Type 4 (*n* = 1), BA/CPAM Type 2 (*n* = 17), intralobar BPS (*n* = 3), and unclassified (*n* = 4).

## Postoperative lung function after thoracotomy

### Comparison of lung function between healthy children and patients

The mean postoperative lung function values in the patients who underwent thoracotomy were as follows: %VC 81.2 ± 16.1%; %FEV_1_ 76.1 ± 17.5%; and FEV_1_/FVC 82.2 ± 10.2%. Lung function data from patients who underwent thoracoscopic surgery (*n* = 3) are presented separately in Supplementary Table 2 because the small sample size precluded meaningful statistical comparisons. %VC and FEV_1_/FVC were within the normal range, but both were significantly lower than values in age-matched healthy controls (both *P* < 0.01). In contrast, %FEV_1_ was below the normal limit and significantly lower than that of healthy controls (*P* < 0.01). A reduced lung function below the normal range was observed in 10 patients (37.0%) for %VC and in 8 patients (29.6%) for FEV_1_/FVC (Table 2).

**Table 2:** Comparisons of the Lung Function Tests Between the Patients and Controls

	Patients		Controls		P value	
n	27		56		NA	
Sex, male	16	(59.3)	31	(55.4)	0.82	
Age at Spirometry, years	12.0	± 2.8	12.5	± 2.4	0.35	
%VC	81.2	± 16.1	91.7	± 12.3	0.001*	
%FEV_1_	76.1	± 17.5	93.3	± 13.0	< 0.001*	
FEV_1_/FVC	82.2	± 10.2	89.4	± 5.9	0.002*	

*P < 0.05 indicates statistical significance.

Continuous variables are presented as the mean ± SD as appropriate. Normality was tested by Shapiro–Wilk.

P values were not calculated for sample size comparisons.

%VC, percent predicted vital capacity. %FEV₁, percent predicted forced expiratory volume in 1 second. FEV₁/FVC, ratio of forced expiratory volume in 1 second to forced vital capacity.

### Factors associated with the lung function after thoracotomy

Associations between the postoperative lung function and clinical variables, including age, prenatal diagnosis, extent of resection, affected side, and long-term morbidities, were evaluated in the patients who underwent thoracotomy (Table 3). Fetal surgery was not included in the univariate analyses because only one patient underwent fetal surgery.

**Table 3:** Comparisons of the Lung Function Tests According to the Clinical and Surgical Factors

Age at surgery	n	%VC	%FEV_1_	FEV_1_/FVC	
≤ 28 days	6 (22.2)	68.9 ± 24.4	67.2 ± 31.6	90.4 (82.4–93.6)	
> 28 days	21 (77.8)	84.7 ± 11.4	78.6 ± 10.9	82.7 (74.7–87.6)	
P value		0.03^*^	0.16	0.34	
Prenatal diagnosis	n	%VC	%FEV_1_	FEV_1_/FVC	
+	22 (81.5)	79.9 ± 17.5	75.7 ± 18.8	82.9 ± 10.6	
-	5 (18.5)	86.6 ± 6.1	77.6 ± 11.3	79.5 ± 8.7	
P value		0.42	0.83	0.52	
Extent of resection	n	%VC	%FEV_1_	FEV_1_/FVC	
Lobectomy or more	21 (77.8)	80.2 ± 17.0	78.0 (63.3–85.1)	81.1 ± 10.2	
Less than lobectomy	6 (22.2)	84.5 ± 13.0	78.4 (76.6–82.0)	86.2 ± 10.2	
P value		0.59	0.51	0.29	
Affected side	n	%VC	%FEV_1_	FEV_1_/FVC	
Right	14 (51.9)	81.7 ± 17.6	74.6 ± 20.4	80.4 ± 12.5	
Left	13 (48.1)	80.6 ± 15.0	77.6 ± 14.5	84.3 ± 6.9	
P value		0.87	0.67	0.34	
Asthma-like symptoms	n	%VC	%FEV_1_	FEV_1_/FVC	
+	10 (37.0)	78.3 ± 20.2	67.6 ± 20.7	75.5 ± 12.0	
-	17 (63.0)	82.9 ± 13.5	81.0 ± 13.7	86.3 ± 6.6	
P value		0.48	0.05	0.02*	
Musculoskeletal morbidities	n	%VC	%FEV_1_	FEV_1_/FVC	
+	8 (29.6)	64.9 ± 13.6	58.7 ± 15.9	79.5 ± 13.1	
-	18 (66.7)	89.3 ± 10.0	84.8 ± 10.5	83.7 ± 8.5	
P value		<0.001*	<0.001*	0.32	

*P < 0.05 indicates statistical significance.

Fetal intervention was performed in only one patient in the thoracotomy cohort; therefore, no statistical comparison was performed.

Continuous variables are presented as the mean ± SD or median (interquartile range, IQR) as appropriate. Normality was tested by Shapiro–Wilk, and between-group comparisons by t-test, Welch’s t-test, or Mann–Whitney U test, as appropriate.

%VC, percent predicted vital capacity. %FEV₁, percent predicted forced expiratory volume in 1 second. FEV₁/FVC, ratio of forced expiratory volume in 1 second to forced vital capacity.

A univariate analysis showed that a decreased lung function was significantly associated with asthma-like symptoms and musculoskeletal morbidities.

In multivariable linear regression (Table 4);

**Table 4:** Factors Associated with the Lung Function Tests

Variables		n	Estimate	B	Standard Error	T	P	95% Confidence Interval			Vif	
								Lower	Upper			
%VC	Intercept		89.288		2.655	33.634	<0.001	88.8		94.8		
	Musculoskeletal morbidities	9	−24.355	−0.727	4.599	−5.287	<0.001	−33.8		−14.9		NA
	adjusted R^2^=0.510, P<0.001*											
%FEV_1_	Intercept		88.362		3.111	28.400	<0.001	81.9		94.8		
	Asthma-like symptoms	10	−10.803	−0.304	4.604	−2.347	0.028	−20.3		−1.3		1.012
	Musculoskeletal morbidities	9	−24.878	−0.682	4.716	−5.275	<0.001	−34.6		−15.1		1.012
	adjusted R^2^=0.570, P<0.001*											
FEV_1_/FVC	Intercept		86.259		2.159	39.953	0.001	81.8		90.7		
	Asthma-like symptoms	10	−10.899	−0.303	3.548	−3.044	0.005	−18.1		−3.5		NA
	adjusted R^2^=0.241, P=0.005*											

P values for the regression models indicate overall model significance.

* P < 0.05 indicates statistical significance.

B indicates standardized regression coefficients.

n indicates the number of patients with the specified condition included in the model.

%VC, percent predicted vital capacity. %FEV₁, percent predicted forced expiratory volume in 1 second. FEV₁/FVC, ratio of forced expiratory volume in 1 second to forced vital capacity.

- %VC was independently associated with musculoskeletal morbidities (adjusted R^2^=0.510, *P* < 0.001).
- %FEV_1_ was independently associated with asthma-like symptoms and musculoskeletal morbidities (adjusted R^2^ = 0.570, *P* < 0.001).
- FEV_1_/FVC was independently associated with asthma-like symptoms (adjusted R^2^ = 0.241, *P* = 0.005).

No multicollinearity was detected (all VIF < 1.5).

Exploratory analyses showed no significant associations between postoperative lung function and skin incision length or the intercostal level of thoracotomy (all *p* > 0.05).

### Sensitivity analysis in patients undergoing lobectomy

A sensitivity analysis was performed on patients who underwent single-lobe resection (*n* = 20). The results were consistent with those of the overall cohort; lung function remained lower than that of healthy controls. In multivariate analyses, musculoskeletal deformities remained associated with %VC and %FEV_1_, whereas asthma-like symptoms were not retained as independent predictors of %FEV_1_. The detailed lung function values and regression results for this subgroup are provided in Supplementary Table 3.

### Longitudinal changes in the lung function

Table 5 shows the longitudinal changes in lung function tests. Fourteen patients had more than 1 spirometry assessment (median follow-up duration, 3.5 [3.0–5.0] years). No significant changes were observed in %VC, % FEV_1_, or FEV_1_/FVC over time.

**Table 5:** Longitudinal Changes in the Lung Function Tests

	First examination	Latest examination	P value	
n	14	14	NA	
Age at test, years	7.9 (7.2–9.0)	12.2 (11.0–13.0)	NA	
%VC	81.6 ± 12.0	84.3 ± 14.6	0.58	
%FEV_1_	77.0 ± 11.6	81.7 ± 11.7	0.30	
FEV_1_/FVC	87.4 ± 8.1	83.4 ± 8.2	0.19	

Continuous variables are presented as the mean ± SD or median (interquartile range, IQR) as appropriate. Normality was tested by Shapiro–Wilk

Paired comparisons between the first and latest examinations were performed using the paired t-test or Wilcoxon signed-rank test, as appropriate.

P values were not calculated for age at the time of testing because this variable differs by definition between the two time points.

%VC, percent predicted vital capacity. %FEV₁, percent predicted forced expiratory volume in 1 second. FEV₁/FVC, ratio of forced expiratory volume in 1 second to forced vital capacity.

## Pulmonary and musculoskeletal morbidities

Table 6 shows long-term pulmonary and musculoskeletal morbidities. The long-term morbidities in patients who underwent thoracoscopic surgery are summarized in Supplementary Table 4. Asthma-like symptoms were reported in 10 patients (37.0%), with 7 receiving medical treatment. At the time of the survey, six patients (22.2%) had ongoing, predominantly mild symptoms; four of these patients were in late adolescence or older.

**Table 6:** Long-term Morbidities (n=27)

Asthma-like symptoms	10	(37.0)
Musculoskeletal morbidities	9	(33.3)
Chest wall deformities^a^	8	(29.6)
Scoliosis^b^	4	(21.1)

^a^One patient had both pectus excavatum and some other chest wall deformity.^b^Based on the 19 patients who consented to spinal radiography.

Data are presented as n (%).

Nine (33.3%) patients developed postoperative chest or spinal deformities. Pectus excavatum was observed in five patients (median Haller Index: 7.68 [4.84–9.49]), all of whom underwent corrective surgery. Four patients had other chest wall deformities, including unilateral or asymmetric lateral depression, unilateral shoulder elevation, or breast asymmetry. One patient exhibited both pectus excavatum and another form of chest wall deformity. Among 19 patients who underwent spinal radiography, scoliosis was identified in 4 (21.1%), and 2 required orthopedic interventions. Two patients had scoliosis and chest wall deformities. Most deformities did not limit the exercise capacity. Patients with postoperative musculoskeletal morbidities showed no statistically significant associations with skin incision length or intercostal thoracotomy level in the logistic regression or Fisher’s exact test analyses.

## Quality of life

Table. 7 Twenty-five patients (92.6%) completed PedsQL™. A total of 25 patients and 31 healthy controls were included in this study. No significant differences in overall HRQOL scores were found in either the self-report or parent-proxy questionnaires (Table 7). There was a significant age difference between the two groups; however, age-appropriate questionnaires were used for this assessment to minimize the potential impact of age differences on the results. HRQOL data for patients who underwent thoracoscopic surgery (*n* = 3) are presented separately in Supplementary Table 5 owing to the small sample size.

**Table 7:** Comparisons of the Quality of Life Questionnaire Between the Patients and Controls

	Patients	Controls	P value
n	25	31	NA
Sex, male	15 (60.0)	21 (67.7)	0.84
Age at test, years	14.1 ± 4.5	11.1 ± 2.5	0.02*
Child Self-Report^a^	98.0 (91.4–100.0)	93.8 (88.0–98.8)	0.16
Parent Proxy Report^a^	97.5 (92.2–100.0)	96.3 (92.7–99.1)	0.42

*Age-appropriate questionnaires were used according to the survey design; therefore, differences in chronological age do not affect the scoring.

^a^Self- and parent-proxy questionnaires assessed physical, emotional, social, and school functioning scores, which were summed and compared between patients and healthy controls.

Data are presented as n (%), the mean ± SD or median (interquartile range, IQR).

## Discussion

In this retrospective cohort study, we evaluated the long-term lung function, musculoskeletal morbidities, and HRQOL in children undergoing thoracotomy lung resection for congenital lung malformations.

Regarding the lung function, %VC and FEV_1_/FVC were within normal limits, but were significantly lower than those in age-matched controls. In contrast, %FEV_1_ was below the normal range and significantly lower than that in controls. The multivariate analysis identified asthma-like symptoms and musculoskeletal morbidities as independent predictors of reduced lung function. No significant longitudinal changes in the lung function were observed. Mild asthma-like symptoms occurred in 37.0% of patients, and some symptoms persisted even in older adolescents. Moreover, musculoskeletal morbidities were observed in 33.3% of the patients, with a subset requiring surgical correction.

Our findings that %VC and FEV_1_/FVC remained within the normal limits are consistent with previous studies that reported preserved lung volumes after pediatric lobectomy [7,14]. However, when compared with healthy controls, our results showing reduced lung function tests indicate that lung resection in childhood may have a long-term impact on the lung function. The abnormal %FEV_1_ is consistent with previous reports linking reduced expiratory flow to airway hyperreactivity or altered airway mechanics [7,8].

The incidence of musculoskeletal morbidities in our cohort (33.3%) was comparable to the 15–37% reported in a previous pediatric thoracotomy series [15, 16]. Notably, all cases of severe pectus excavatum required surgical correction, whereas milder deformities and scoliosis were generally well tolerated.

Previous studies comparing thoracotomy and thoracoscopic approaches have suggested that thoracoscopic surgery may be associated with better musculoskeletal outcomes and preserved lung function in children with congenital lung malformations; however, these findings have not been consistent across studies [6, 9]. In the present study, a direct comparison between the surgical approaches was not feasible because only a small number of patients underwent thoracoscopic surgery. Accordingly, the analyses were restricted to patients who underwent thoracotomy to allow a more homogeneous evaluation of long-term outcomes.

Within this thoracotomy cohort, postoperative musculoskeletal morbidities were consistently associated with reduced lung function, supporting previous reports on the adverse impact of chest wall deformities on long-term respiratory mechanics [6]. The timing of surgery was explored in relation to the long-term lung function. Within the thoracotomy cohort, surgery performed within the neonatal period (≤ 28 days of age) was associated with a lower %VC in the univariate analysis; however, this finding should be interpreted with caution because only six patients underwent surgery in the neonatal period. The surgical timing was not retained in the multivariate analyses. In subgroup analyses limited to patients who underwent single-lobe resection, no clear association was observed between surgical timing and %VC, %FEV_1_, or FEV_1_/FVC. Given the extremely limited number of neonatal cases, definitive conclusions regarding the impact of surgical timing on long-term lung function cannot be drawn. In contrast, neither the extent of resection nor lesion laterality showed significant associations with lung function in univariate analyses and, therefore, were not retained as independent predictors in multivariable models. Comparable findings were observed in subgroup analyses that were limited to patients who underwent single-lobe resection.

Exploratory analyses focusing on thoracotomy-specific factors, including skin incision length and intercostal level, demonstrated no significant associations with postoperative lung function or prevalence of musculoskeletal morbidities. Therefore, these parameters are unlikely to represent independent determinants of long-term outcomes, and may instead reflect technical or anatomical considerations at the time of surgery. These findings should be interpreted cautiously given the limited sample size and the potential influence of compensatory lung growth during childhood, during which alveolar and pulmonary vascular development continues into late childhood. 

In analyses restricted to single-lobe resection, the association between asthma-like symptoms and lung function was attenuated, likely reflecting the reduced statistical power.

Despite remaining within the normal limits, reduced %VC and FEV_1_/FVC may result from the combined effects of parenchymal loss and subtle mechanical restriction from chest wall deformities. Reduced %FEV_1_ reflects both airway caliber reduction and hyper-reactivity.

Long-term follow-up is particularly important in children who exhibit airway hyperreactivity or musculoskeletal deformities. These patients may benefit from early pulmonary rehabilitation, bronchodilator therapy if indicated, and regular musculoskeletal assessments. Despite these measurable deficits, most children maintain good exercise tolerance and HRQOL, and their subjective symptoms do not necessarily reflect an impaired respiratory function.

This study is limited by its retrospective design, small sample size, and the exclusion of patients without lung function data. The control group was hospital-based and imaging-based volumetric lung assessment was not performed. The HRQOL was evaluated cross-sectionally and may not reflect longitudinal changes. Additionally, the limited number of thoracoscopic cases precluded meaningful comparisons between surgical approaches.

## Conclusion

After lung resection for congenital lung malformations by thoracotomy, %VC and FEV_1_/FVC remained within the normal limits, but were significantly lower than those of age-matched controls, while %FEV_1_ was frequently abnormal. Airway hyper-reactivity and musculoskeletal deformities are associated with a reduced lung function.

Postoperative respiratory symptoms and musculoskeletal deformities suggest a decline in the respiratory function. Comprehensive long-term follow-up and therapeutic interventions are recommended for these patients.

## Supplementary Information

Below is the link to the electronic supplementary material.

Supplementary material 1 (DOCX 30.1 kb)

Supplementary material 2 (DOCX 29.1 kb)

Supplementary material 3 (DOCX 33.3 kb)

Supplementary material 4 (DOCX 28.7 kb)

Supplementary material 5 (DOCX 29.7 kb)

## Data Availability

The authors declare that all data in this manuscript are available within the article.
