# Supplementary material for: Long-term Outcomes of Children Undergoing Thoracotomy Lung Resection for Congenital Lung Malformations
Source: Surg Today. 2026 Feb 11;56(8):1461–9. doi: 10.1007/s00595-026-03242-y (PMC13379478; doi:10.1007/s00595-026-03242-y)
Supplement: Supplementary file 1 — Supplementary material 1 (DOCX 30.1 kb) [file 595_2026_3242_MOESM1_ESM.docx]

| **Supplementary Table 1.** **Patient Background and Surgical Treatments in Patients Who Underwent Thoracoscopic Surgery (n = 3).** | | | | |
| --- | --- | --- | --- | --- |
| Age at surgery, days | | 259.0 | (196.0-349.5) |  |
| Age at follow-up visit, years | | 10.2 | (8.3-10.4) |  |
| Follow-up period, years | | 9.5 | (7.3-9.9) |  |
| Prenatal diagnosis | | 2 | (66.7) |  |
| Fetal intervention | | 1 | (33.3) |  |
| Extent of resection | Lobectomy | 3 | (100.0) |  |
| Affected site, right | | 3 | (100.0) |  |
| Due to the small sample size, no statistical comparisons were performed.  Data are presented as n (%) or the median (interquartile range, IQR). | | | |  |
