# Supplementary material for: Long-term Outcomes of Children Undergoing Thoracotomy Lung Resection for Congenital Lung Malformations
Source: Surg Today. 2026 Feb 11;56(8):1461–9. doi: 10.1007/s00595-026-03242-y (PMC13379478; doi:10.1007/s00595-026-03242-y)
Supplement: Supplementary file 2 — Supplementary material 2 (DOCX 29.1 kb) [file 595_2026_3242_MOESM2_ESM.docx]

| **Supplementary Table 2** **Pulmonary Function Tests in Patients Who Underwent Thoracoscopic Surgery (n = 3).** | |  |
| --- | --- | --- |
| %VC | 85.9 (75.4-86.1) |  |
| %FEV_1_ | 85.1 (67.2-85.6) |  |
| FEV_1_/FVC | 86.5 (78.3-88.2) |  |
| Data are presented as the median (interquartile range, IQR). Due to the small sample size, no statistical comparisons were performed.  %VC, percent predicted vital capacity. %FEV₁, percent predicted forced expiratory volume in 1 second.　 FEV₁/FVC, ratio of forced expiratory volume in 1 second to forced vital capacity. | | |
