# Supplementary material for: Long-term Outcomes of Children Undergoing Thoracotomy Lung Resection for Congenital Lung Malformations
Source: Surg Today. 2026 Feb 11;56(8):1461–9. doi: 10.1007/s00595-026-03242-y (PMC13379478; doi:10.1007/s00595-026-03242-y)
Supplement: Supplementary file 3 — Supplementary material 3 (DOCX 33.3 kb) [file 595_2026_3242_MOESM3_ESM.docx]

| **Supplementary Table 3.** **Factors Associated with Lung Function Tests in Patients Who Underwent Thoracotomy with Single-Lobe Resection (n = 20).** | | | | | | | | | | |
| --- | --- | --- | --- | --- | --- | --- | --- | --- | --- | --- |
|  | Variables | n | Estimate | B | Standard  Error | T | P | 95% Confidence Interval | | |
|  |  |  |  |  |  |  |  | Lower | Upper | |
| %VC | Intercept |  | 91.694 |  | 2.929 | 31.302 | <0.001 | 85.540 | | 97.848 |
|  | Musculoskeletal morbidities | 8 | -24.822 | -0.696 | 4.632 | -5.359 | <0.001 | -34.553 | | -15.092 |
|  | adjusted R^2^=0.593, P<0.001* | | | | | | | | | |
| %FEV1 | Intercept |  | 85.921 |  | 3.734 | 23.013 | <0.001 | 78.078 | | 93.765 |
|  | Musculoskeletal morbidities | 8 | -25.398 | -0.712 | 5.903 | -4.302 | <0.001 | -37.800 | | -12.996 |
|  | adjusted R^2^=0.480, P<0.001* | | | | | | | | | |
| FEV1/FVC | Intercept |  | 86.015 |  | 2.266 | 37.961 | <0.001 | 81.255 | | 90.776 |
|  | Asthma-like symptoms | 7 | -13.887 | -0.379 | 3.830 | -3.626 | 0.002 | -21.933 | | -5.840 |
|  | adjusted R^2^=0.390, P=0.002* | | | | | | | | | |
| P values for the regression models indicate overall model significance.  * P < 0.05 indicates statistical significance.  B indicates standardized regression coefficients.  n indicates the number of patients with the specified condition included in the model.  This analysis was limited to patients who underwent thoracotomy with anatomical single-lobe resection (n=20). Variables not retained in the final model are not shown.  %VC, Percent predicted vital capacity. %FEV₁, Percent predicted forced expiratory volume in 1 second. FEV₁/FVC, Ratio of forced expiratory volume in 1 second to forced vital capacity. | | | | | | | | | | |
