# Supplementary material for: Long-term Outcomes of Children Undergoing Thoracotomy Lung Resection for Congenital Lung Malformations
Source: Surg Today. 2026 Feb 11;56(8):1461–9. doi: 10.1007/s00595-026-03242-y (PMC13379478; doi:10.1007/s00595-026-03242-y)
Supplement: Supplementary file 4 — Supplementary material 4 (DOCX 28.7 kb) [file 595_2026_3242_MOESM4_ESM.docx]

| **Supplementary Table 4. Long-term Morbidities in Patients Who Underwent Thoracoscopic Surgery (n = 3).** | |
| --- | --- |
| Asthma-like symptoms | 1 (33.3) |
| Musculoskeletal morbidities | 1 (33.3) |
| Data are presented as n (%).  No statistical comparisons were performed due to the small sample size.  The musculoskeletal morbidity observed in this cohort was classified as some other chest wall deformity. | |
