# Supplementary material for: Long-term Outcomes of Children Undergoing Thoracotomy Lung Resection for Congenital Lung Malformations
Source: Surg Today. 2026 Feb 11;56(8):1461–9. doi: 10.1007/s00595-026-03242-y (PMC13379478; doi:10.1007/s00595-026-03242-y)
Supplement: Supplementary file 5 — Supplementary material 5 (DOCX 29.7 kb) [file 595_2026_3242_MOESM5_ESM.docx]

| **Supplementary Table 5. Quality of Life Scores in Patients Who Underwent Thoracoscopic Surgery (n = 3)** | |
| --- | --- |
| Sex, male | 3 (100.0) |
| Age at test, years | 10.1 (8.2-10.2) |
| Child Self-Report^a^ | 88.4 (84.7-91.2) |
| Parent Proxy Report^a^ | 93.8 (93.4-93.8) |
| ^a^ Self- and parent-proxy questionnaires assessed physical, emotional, social, and school functioning scores, which were summed and compared between patients and healthy controls.  Data are presented as n (%) and the median (interquartile range, IQR). | |
